# Two-step synthesis of millimeter-scale flexible tubular supercapacitors

**DOI:** 10.1038/s42004-020-0272-7

**Published:** 2020-02-21

**Authors:** Chao Lu, Xi Chen

**Affiliations:** 1 Department of Earth and Environmental Engineering, Columbia University, New York, NY 10027 USA; 2 School of Chemical Engineering, Northwest University, Xi’an, Shaanxi 710069 China

**Keywords:** Devices for energy harvesting, Supercapacitors, Materials for devices, Materials for energy and catalysis, Soft materials

## Abstract

Flexible supercapacitors have been demonstrated to be ideal energy storage devices owing to their lightweight and flexible nature and their high power density. However, conventional film-shaped devices struggle to meet the requirements of application in complicated situations, including medical instruments and wearable electronics. Here we report a hollow-structured flexible tubular supercapacitor prepared from a scalable method with the same diameter as electric wires. This new supercapacitor design allows for a large specific capacitance of 102 F g^−1^ at a current density of 1 A g^−1^ with excellent air-working stability over 10,000 cycles. It also shows a high energy density of 14.2 Wh kg^−1^ with good rate capability even at a current density of 10 A g^−1^, which is superior to commercial devices (3–10 Wh kg^−1^). Moreover, the device delivers a stable energy storage capacity when encountering different flexible conditions, such as elongated, tangled and bent states, showing wide potentials in flexible and even wearable applications. Especially, it retains stable specific capacitance even after 500 bending cycles with a bending angle of 180°. The two-step fabrication method of these flexible tubular supercapacitors may allow for possible mass production, as they could be easily integrated with other functional components, and used in realistic scenarios that conventional film devices struggle to realize.

## Abstract (web summary)

Flexible supercapacitors are versatile energy storage devices owing to their light weight, flexibility and high power density. Here a scalable, flexible, tubular supercapacitor possessing the diameter of an electrical wire is presented.

## Introduction

Over the past decades, flexible supercapacitors have witnessed an enormous development owing to the dramatic innovation of wearable electronic devices, which require to work in close contact with the human body^1–3^. It is critical to develop flexible supercapacitors with fast charging rate, high energy capacity, and long life span, as well as good mechanical properties that allows their practical applications under flexible conditions^4–6^. Comparing with conventional battery systems, the major challenge impeded applications of flexible supercapacitors is their relatively lower energy density, which is mainly controlled by intrinsic properties of the electrode materials^7,8^. To address this issue, tremendous efforts have been made for the purpose of developing high energy density electrode materials, such as conducting polymers^9,10^, CNTs^11^, graphene^12^, Co_3_O_4_^13^, MoS_2_^14^, graphitic carbon nitride^15,16^ and so on. Resultly, these nanostructural electrode materials with good electrical conductivity, high surface area and porous structure greatly improved energy density of supercapacitors to the practical application level competitive to batteries^17,18^. But it is still challenging to fabricate flexible supercapacitors with proper morphology and superior mechanical properties that can accommodate complicated deformations, such as bending, twisting and stretching.

One-dimensional (1D) flexible supercapacitors are promising to meet the requirements of mechanical properties for wearable applications^18,19^. Compared with two-dimensional (2D) and three-dimensional (3D) devices, 1D devices are more flexible to accommodate deformations and can even be woven with textiles and fabrics with different materials that fit the different curved targeted surfaces^20,21^. For instance, a 1D supercapacitor based on electrospinning technique was reported with high energy density and good flexible energy storage properties^22^. Another coaxial asymmetric 1D supercapacitors prepared with electrical machinery was reported with a high volumetric energy density and ultrafast charging rates^23^. Recently, wearable asymmetric fiber-shaped supercapacitors have extensively investigated based on various nanomaterials, including MOF-derived carbon nanomaterials^24^, core-shell nanomaterials^25^, and VN/CNTF^26^. The fiber supercapacitors all display excellent flexibility, superior integratability, long life and high energy density, and present great potential as power sources for wearable applications. These previously reported devices showed high energy storage capacity and flexible performances for potential applications, but the fabrication processes of supercapacitors always referred to complicated materials synthesis methods and relied on advanced instruments and equipment. The drawbacks made mass-production of flexible devices impossible and seriously hindered their practical applications^27,28^. Thus it is urgent to put forward a simple and effective fabrication method of 1D flexible supercapacitors without complicated materials and large-scale instruments.

In this work, we report a high-performance flexible tubular supercapacitor prepared through a simple and scalable strategy. The as-fabricated 1D device with a diameter of about 3 mm is very different from conventional 2D film devices, as its hollow structure makes it suitable for integration with other electrical devices in various application scenarios. It exhibits a large specific capacitance of 102 F g^−1^ at the current density of 1 A g^−1^ and retains a stable energy storage capacity even after working 10000 times in air. The high rate capability from 1 to 10 A g^−1^ makes its applications under high charging rates possible, which satisfies the requirements of fast charge for energy storage systems in electronic products. The achieved energy density is as high as 14.2 Wh kg^−1^ and is superior to that of commercial capacitors (3–10 Wh kg^−1^). It is noteworthy that this tubular supercapacitor delivers a stable energy storage capacity under different flexible conditions, including elongation, tangle and bending, showing wide potentials in flexible and even wearable applications. Its specific capacitance retention remains at 92% after 500 bending cycles under a bending angle of 180°. Moreover, the simple and cost-effective fabrication strategy would make mass production of flexible supercapacitors possible and could promote their practical applications in future. This work presents an insight into the morphology and design of 1D flexible energy devices and hopes to accelerate the development of other flexible energy storage systems, such as in solar cell, fuel cell, and lithium battery applications.

## Results and discussion

### Fabrication of flexible tubular supercapacitors

**Fig. 1:**
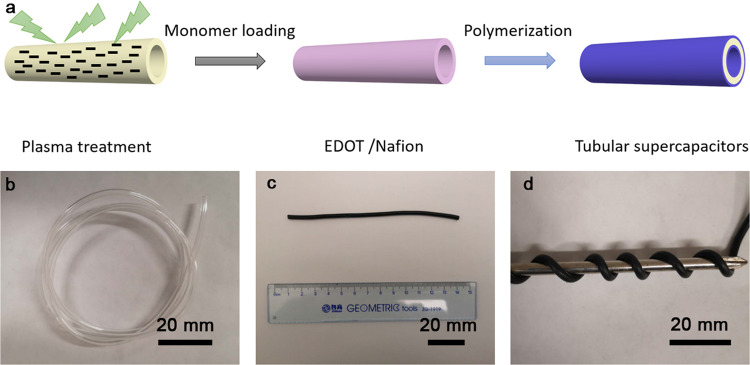
Fabrication of flexible tubular supercapacitors. **a** Schematic for fabrication process. Optical images of **b** Nafion tubes, **c** the as-fabricated device, **d** flexible device tangled on an iron rod.

The two-step fabrication method of flexible tubular supercapacitors was illustrated in Fig. 1a. Firstly, the Nafion tube was pretreated by plasma etching to obtain a rough surface for adsorbing more monomers (EDOT, 3,4-ethylenedioxythiophene). The rough surface could also help to form robust interface layers between electrode and electrolyte, which is critical to flexibility of supercapacitors. Then, the Nafion tube was soaked with EDOT solution in order to form Nafion/EDOT composite layer on inner and outer surfaces of Nafion tube. Lastly, the Nafion/EDOT tube was immersed into FeCl_3_ solution for polymerization of PEDOT (poly(3,4-ethylenedioxythiophene)) electrode. Chemical structures of Nafion and PEDOT materials are presented in Supplementary Fig. 1. The fabrication process of device does not involve with any large-scale equipments or other materials with complicated structure, which is promising for its mass-production in future. The optical images of transparent Nafion tube and as-prepared tubular supercapacitor were shown in Fig. 1b, c. The electrolyte (Nafion) and electrode (PEDOT) layers are both made from soft polymers, which determine the superior mechanical properties of tubular supercapacitors. Structural characterization data, including XRD and Raman characterizations, have been carried out and shown in Supplementary Fig. 2. In XRD pattern of Nafion, two wide peaks centered at 17° and 39°, which are characteristic peaks of the perfluorocarbon backbone of Nafion^29,30^. From the Raman spectra of PEDOT materials, the sharp peak visible at 1429 cm^−1^ is attributed to the characteristic symmetric Cα=C_β_ (–O) stretching^31^. The broad peak at 1505 cm^−1^ represents asymmetric C=C stretching mode of PEDOT^32^. The medium and weak bands near 437, 573, 699, 988, 1110, and 1261 cm^−1^ are likewise typical for PEDOT^33^. It is found that the device can be twisted around an iron rod, showing its excellent flexibility (Fig. 1d). The good flexibility makes it promising for integration with many flexible electric devices in different flexible conditions. Comparing to carbon nanomaterials, including graphene and carbon nanotubes, PEDOT is not only cost-effective, but also easy to integrate with Nafion owing to its intrinsic flexibility and the compatibility with polymer electrolyte. This work is mainly about the tubular structure design for integration with macroscopic electrical devices, which cannot be realized by micro/nano-devices like fiber devices. Moreover, the tubular supercapacitor is not designed in the range of micro/nano-device because it is applied to integrate with other tubular electrical devices for specific applications, such as medical catheters, tubular robots, and unmanned aerial vehicle.

### Morphology of flexible tubular supercapacitors

**Fig. 2:**
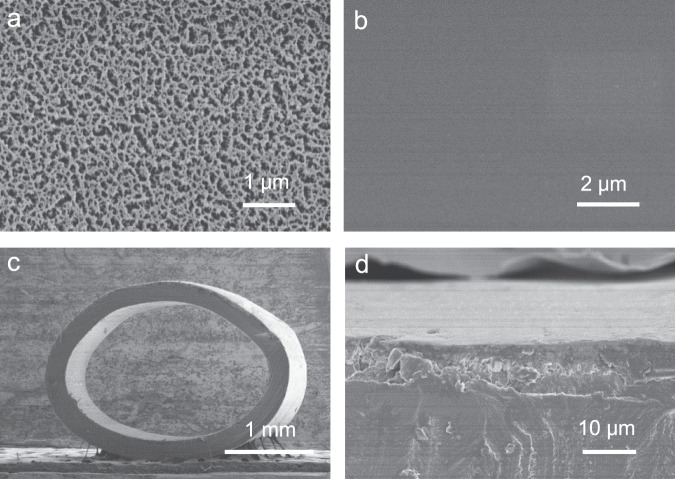
Morphology of flexible tubular supercapacitors. SEM images of **a** plasma treated surface of Nafion tube, **b** surface of supercapacitors, **c** cross-sectional image of the device, **d** interface structure of the device.

Commercial Nafion tube was etched with plasma method to obtain a rough surface, which is vital for the next-up polymerization process of PEDOT electrodes. Previous studies found that physical etching treatment did not alter the chemical structure of materials and thus performance of the device^34^. Figure 2a displays the rough surface of plasma treated Nafion tube with many microcracks, which can accommodate more EDOT monomers and promote the formation of robust PEDOT electrodes afterward. After finishing polymerization process, the rough surface of Nafion was covered with tight and smooth PEDOT layers, as shown in Fig. 2b. The low-resolution SEM image in Fig. 2c displays the cross-sectional morphology of tubular supercapacitors. It is found that the device shows a hollow structure with two electrode layers distributed inner and outer surface of polyelectrolyte tube. The outside diameter and thickness of the device are 3 mm and 214 μm, respectively. High-resolution SEM image in Fig. 2d presents interface structure of the device. And the thickness of PEDOT electrode layer was found to achieve as high as 13 μm. The robust coupling interface between PEDOT and Nafion layers is beneficial to flexibility of the device because the interface provides sites for materials and energy exchange during electrochemical charge-discharge processes, especially under flexible conditions^35^. N_2_ adsorption-desorption isotherm and the pore size distribution curve of the as-prepared sample have been measured and shown in Supplementary Fig. 3. Specific surface area of the sample is 24 m^2^ g^−1^, and the pore size is mainly distributed around mesopores (centered at 8 nm) and macropores (20–90 nm) range. The porous structure with high surface area will facilitate ion transfer kinetics in the electrochemical devices.

### Electrochemical properties of flexible tubular supercapacitors

**Fig. 3:**
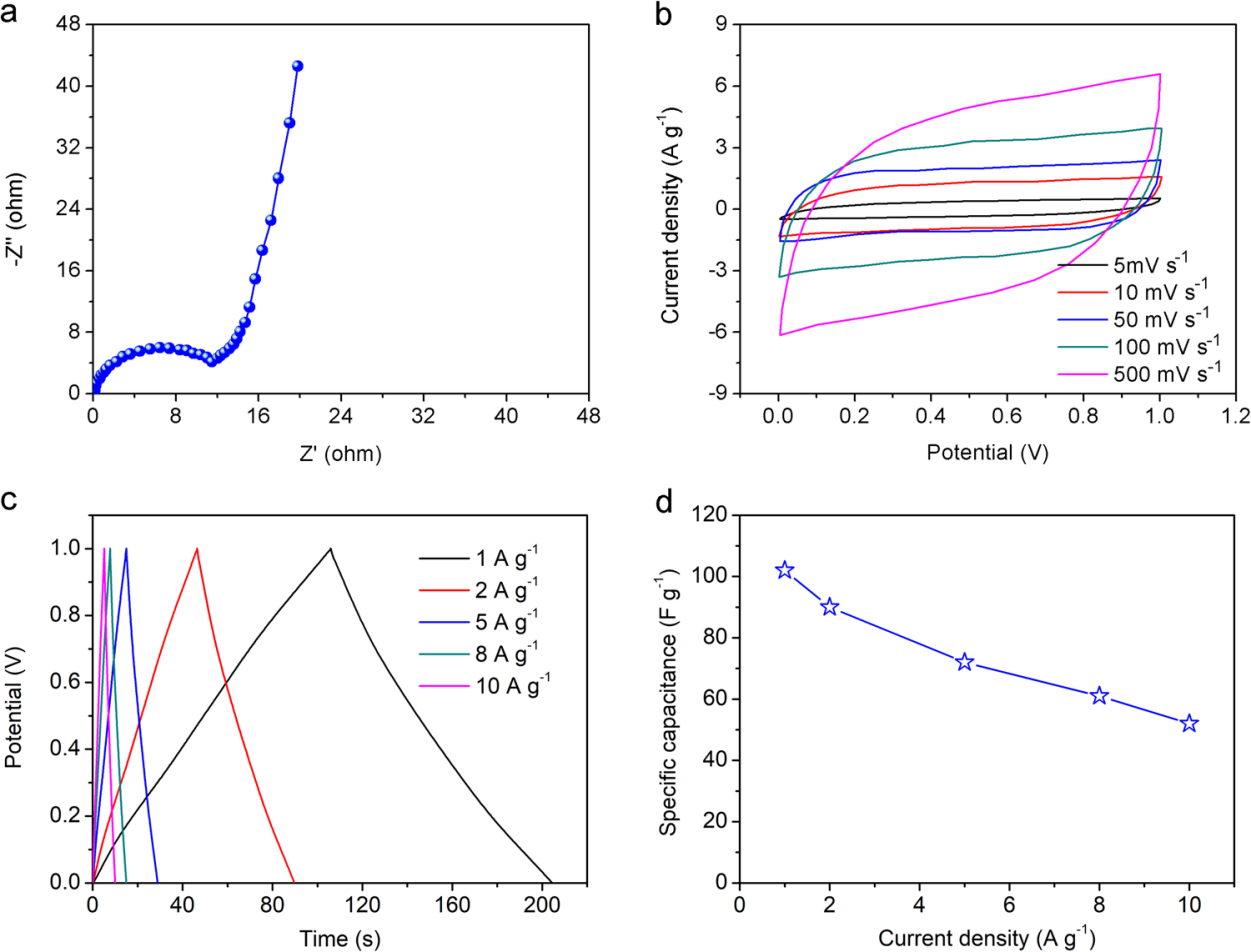
Electrochemical properties of flexible tubular supercapacitors. **a** Nyquist plot for the device. **b** CV curves of devices at different current densities. **c** GCD curves of devices at different current densities. **d** Specific capacitance variation of the devices as function of current densities.

Working mechanism of tubular supercapacitor is illustrated in Supplementary Fig. 4. Nafion is a kind of cation exchange resin with mobile hydrated cations and immobile anions fixed on polymer structure. Only cations immigrate to cathode during charge-discharge process and the single ion immigration mechanism improves ion mobility so as to promote the electrochemical dynamics^36,37^. Nyquist plot of the device is shown in Fig. 3a and the corresponding equivalent circuit model is presented in Supplementary Fig. 5. Its equivalent series resistance is 11.5 Ω, verifying good interfacial contact and electronic conductivity. The hollow structure is designed for integration with macroscopic electrical devices, which cannot be realized by micro/nano-devices like fiber devices. This supercapacitor should be taken as energy storage shell for electrical devices with visible electrical devices, thus energy capacity of the device would be better to evaluate with mass energy density of the device rather than volume energy density. Figure 3b shows cyclic voltammograms (CV) curves of the device under scan rates from 5 to 500 mV s^−1^ and the rectangular shapes verify capacitive mechanism. Galvanostatic charge-discharge (GCD) curves of the supercapacitor are obtained under various current densities from 1 to 10 A g^−1^ in Fig. 3c to calculate its specific capacitances. Their approximate triangular shapes prove the good coulombic efficiency. It is found that capacitance of tubular supercapacitor is 102 F g^−1^ at the current density of 1 A g^−1^ and achieves at 57 F g^−1^ at the high current density of 10 A g^−1^. These results indicate that the tubular supercapacitors get high energy storage capacity and good rate capability.

### Cycling performance of flexible tubular supercapacitors

**Fig. 4:**
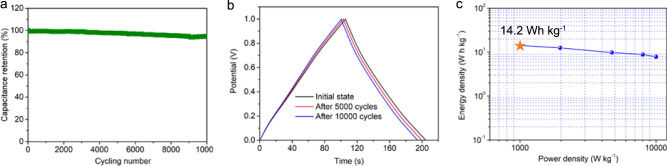
Cycling stability and energy density of flexible tubular supercapacitors. **a** Working cycles of the devices. **b** GCD curves at different status. **c** Ragone plot of the devices.

Additionally, long-term working stability of tubular supercapacitor was evaluated in Fig. 4a by galvanostatic charge-discharge test at the current density of 1 A g^−1^ within potential window of 1.0 V. It is found that the device shows only a slight degradation of specific capacitance after 10,000 working cycles in air. As shown in Fig. 4b, long term specific capacitance of the device remains at 94.5% of its initial value, displaying excellent cycling stability for practical applications. Ragone plot for the supercapacitor in Fig. 4c indicates that its energy density is about 14.2 Wh kg^−1^ at a current density of 1 A g^−1^, which exceed to that of commercially available devices (mainly 3–10 Wh kg^−1^)^3,38^ and achieved relative high-level energy storage capacity of flexible supercapacitor. These results manifest that the tubular supercapacitor can reach high energy densities with excellent air-working stability.

To clarify the reason for stable electrochemical performance of the device, we have characterized the device interface with SEM method at higher magnification. As display in Supplementary Fig. 6a, the PEDOT electrode layer formed tight interface with Nafion polyelectrolyte layer without any obvious microcracks. In order to further characterize structural information and chemical states of the device interface, we scrape off the PEDOT electrode layer from the device and then measured the exposed interface utilizing XPS equipment. As shown in Supplementary Fig. 6b, the S2p spectra of device interface can be fitted into four peaks. The two positions at 163.9 and 165.0 eV are attributed to S2p_3/2_ and S2p_1/2_ characteristic peaks for –C-S-C– covalent bond of thiophene-type sulfur owning to the spin-orbit splitting^39^. The other two positions at 166.0 and 168.3 eV are assigned to –C-S(O)_2_-C– sulfone bridges^40^. These characteristic peaks all belong to PEDOT materials and do not exist in Nafion materials. The results verify that the device interface contains PEDOT materials, which indicates that the PEDOT electrode layer forms tight interface coupling with Nafion polyelectrolyte layers (as shown in the inset) and thus leads to stable electrochemical performance of device.

### Flexibility and integration property of flexible tubular supercapacitors

Mechanical performances of tubular supercapacitors determine whether they can be applied and integrated with flexible and even wearable electronic devices in practical applications. Flexible energy storage performances of the device were investigated using electrochemical charge-discharge method under different deformation conditions, including bending and twisting. The tubular supercapacitor is designed to integrate with other tubular electrical devices for specific applications, such as medical catheters, tubular robots, and unmanned aerial vehicle. But the widely studied fiber-shaped solid-wire devices with micro/nano sizes can not realize such integration property with macroscopic devices. This tubular supercapacitor is made with polymer electrode and electrolyte, which are both flexible enough to endure external forces/stresses. The compatibility of two polymer layers would be beneficial to robust interface of the device, which guarantees the stable energy storage property under flexible conditions. On the one hand, graphene and CNT fibers are much more expansive than PEDOT electrode and not easy to integrate onto solid-state polymer electrolytes because of modulus mismatch of organic and inorganic materials^41,42^.

**Fig. 5:**
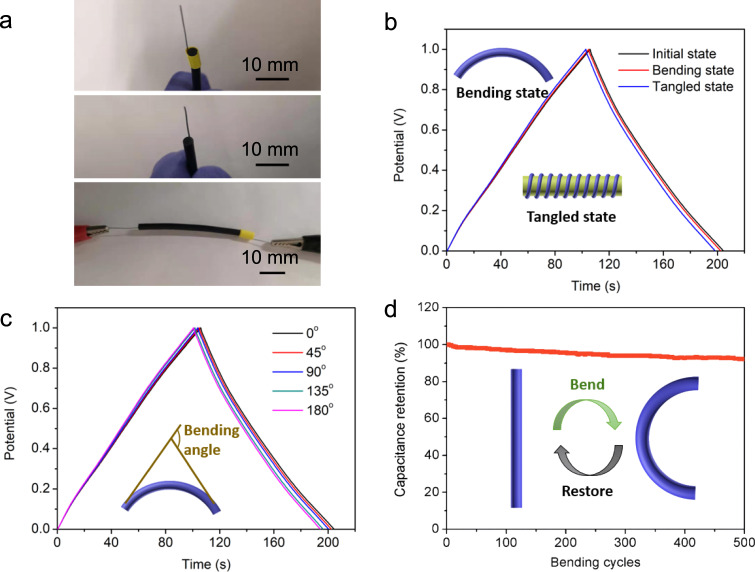
Flexibility of tubular supercapacitors. **a** Optical image of connecting way of the device for electrochemical tests. **b** GCD curves of the device under different states. **c** GCD curves of the device under different bending angles. **d** Flexible stability of the device. The blue cartoon represents the tubular supercapacitor.

Figure 5a presents optical images of optical images of connecting way of the device for electrochemical tests. As shown in Fig. 5b, GCD curves of the device under bending and tangled states indicate negligible specific capacitance change comparing to the value of initial state. Furthermore, flexible energy storage capacity of the device was tested under different bending angles from 0 to 180° under the current density of 1 A g^−1^ in Fig. 5c. Schematic of inset shows the calculation method of bending angles. The device at bending angle of 180° is presented in Supplementary Fig. 7. The GCD curves verify that this soft device keeps delivering stable energy supply even encountering such large deformations. It is noteworthy to mention that specific capacitance of the device retains as high as 92% after 500 bending cycles, showing its great flexible energy storage performances. Scheme of inset in Fig. 5d shows the cyclic testing method. The good flexibility of tubular supercapacitors mainly attributes to soft properties of electrolyte and electrodes as well as the robust interface structure between them. Niche application of tubular device is to integrate with other tubular electrical devices for specific applications, such as medical catheters, tubular robots, and unmanned aerial vehicle. The thick Nafion tube separate inner and outer electrodes by a much large distance, but Nafion is a highly ion-conductive polyelectrolyte and the active ions during electrochemical processes are mainly distributed near interfaces between electrode and electrolyte^43,44^.

**Fig. 6:**
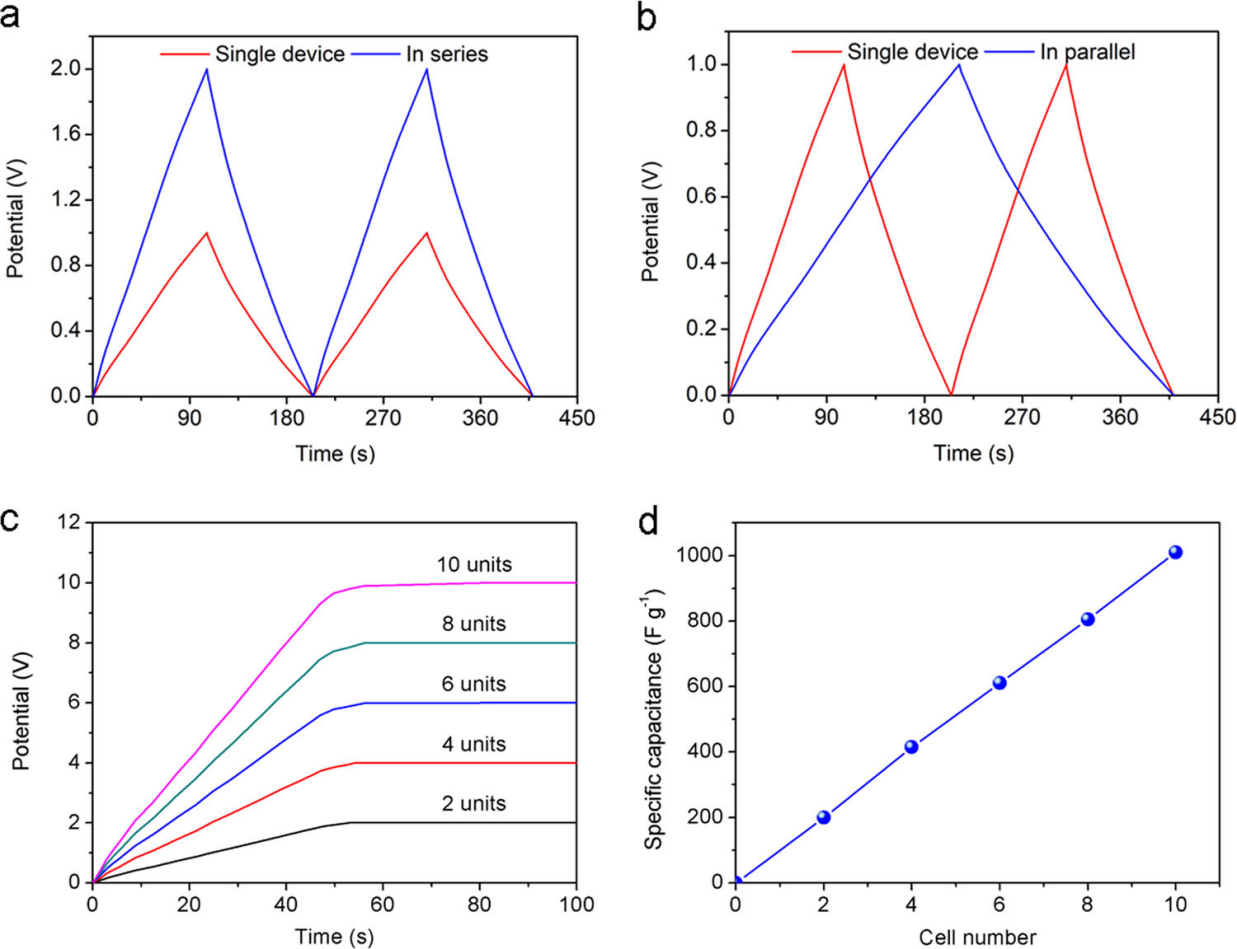
Integration of flexible tubular supercapacitors. GCD curves of two devices connected in **a** series and **b** parallel, respectively. **c** Constant-current charging plots of multi-unit supercapacitors. **d** Relationship between total capacitance and number of devices connected in parallel.

To satisfy voltage and current needs in practical scenarios, sometimes many devices need to integrate in series and parallel and the corresponding equivalent circuit diagrams are displayed in Supplementary Figs. 8 and 9. As shown in Fig. 6a, output voltage of the system increased from 1.0 to 2.0 V when the two devices were connected in series. And specific capacitance of the system increased from 102 to 201 F g^−1^ at current density of 1 A g^−1^ if the two devices were connected in parallel, as displayed in Fig. 6b. The multi-unit system can provide higher voltage and power output by assembling tubular supercapacitors in series. Figure 6c displays constant-current charging curves of the multi-unit system with connected device numbers from 2 to 10 at current density of 1 A g^−1^. Voltage output of the system increases linearly with increasing unit numbers. The multi-unit system can provide higher energy capacity by connecting these units in parallel and its specific capacitance improves linearly with unit numbers in Fig. 6d. We envision that this new concept supercapacitor with good scalability may receive wide applications in microelectronic circuits for wearable or implantable devices. The thermal stability analysis of polyelectrolyte, and effects of humidity and working temperature on electrochemical performances of supercapacitors are displayed in Supplementary Figs. 10–12. The detailed discussions are provided in Supplementary Discussion.

In summary, a tubular supercapacitor with high energy storage capacity and flexibility has been developed by a simple and effective preparation strategy. The proposed two-step preparation method does not involve any complicated material synthesis or large-scale instruments and has potential for scale-up experiments. The as-fabricated supercapacitor with diameter of 3 mm delivers a large specific capacitance of 102 F g^−1^ at the current density of 1 A g^−1^ and displays excellent working stability over 10000 cycles in air. Its energy density achieves as high as 14.2 Wh kg^−1^ with good rate capability and surpasses most of the commercially available devices. The device retains its energy capacity with negligible degradation when encountering flexible conditions, including bending or twisting states. It also kept delivering stable energy supply under bending angle of 180° after 500 cycles, showing wide potential in wearable applications. This work not only paves a way for design of novel stereotype of flexible supercapacitors toward wearable electric devices, but also presents a scalable strategy for mass production of energy storage devices for practical applications.

## Methods

### Materials

Nafion tube was purchased from the HALMA company. Hydrogen peroxide (H_2_O_2_), sulfuric acid (H_2_SO_4_) and Ferric chloride (FeCl_3_) were obtained from Sigma-Aldrich Co., Ltd. 3,4-Ethylenedioxythiophene (EDOT) monomer was purchased from Sinopharm Chemical Reagent Co., Ltd. Deionized water was homemade.

### Fabrication of flexible tubular supercapacitors

Firstly, Nafion tube was pretreated by plasma instrument to make the rough surface. Subsequently, 5%wt hydrogen peroxide and 1 M sulfuric acid solution were applied to wash the Nafion tube to remove residual impurities. Then, the Nafion tube was soaked into EDOT for 1 h and wiped with filter paper. After that, the swollen tube was immersed into 1.5 M FeCl_3_ solution for 0.5 h to complete polymerization. Lastly, the device was washed with methanol and dried at 60 °C under vacuum for 24 h.

### Material characterizations

SEM characterizations were measured with Hitachi S-4800 equipment. Electrochemical performances of the devices were evaluated by Biological electrochemical working station. Mechanical properties of the devices were studied using universal tester (Shimadzu, AGS-X) and motorized translation stages (MTS121).

## Supplementary information

Supplementary Information

## Data Availability

The data that support the findings of this study are available from the corresponding author upon reasonable request.
