# Supplementary material for: Two-step synthesis of millimeter-scale flexible tubular supercapacitors
Source: Commun Chem. 2020 Feb 21;3:23. doi: 10.1038/s42004-020-0272-7 (PMC9814076; doi:10.1038/s42004-020-0272-7)
Supplement: Supplementary file 1 — Supplementary Information [file 42004_2020_272_MOESM1_ESM.pdf]

Supplementary Information

Supplementary Discussion

In order to address the advance and novelty of our study, we have carefully read the referenced literature. He et al. prepared graphene/MnO_2 composite on PET substrate for flexible film-shaped supercapacitors[1]. Gao et al. reported metal-organic framework derived hollow $NiCo_2O_4$ arrays for flexible film-shaped supercapacitors[2]. Chen et al. summarized conducting polymers as electrode materials for flexible film-shaped supercapacitors in that review[3]. Kim et al. reported a flexible yarn-shaped supercapacitor based on carbon nanotube materials[4]. These reported works present advances in development of new electrode materials for flexible supercapacitors, and the devices also show good performances. However, these works all focus on design of novel materials for planar or yarn shaped supercapacitors with the purpose of achieving high energy density and good flexibility, and little attention has been paid on design of novel device shapes, which is very critical to practical integration and application of flexible supercapacitors. The novelty of our study is about the new shape design of tubular supercapacitors, and the materials utilized are common conducting polymers and polyelectrolyte materials without applying complicated structural nanomaterials. The two-step fabrication method made it possible for mass production, and the device can be easily integrated with other functional components for practical application. We have also supplemented some characterizations on materials and device interface to make deep discussion of the underlying high properties, such as XRD, Raman and XPS spectra. It is believed that our study will shed light on design of new device shapes of flexible supercapacitors, and promote their practical applications.

The chemical nature of the employed commercial Nafion tube was described as following. Nafion is a kind of synthetic polymer with ionic properties, such as ion-exchange capacity, ionic conductivity, and hydration capacity[5]. These unique ionic properties of Nafion are based on the tetrafluoroethylene backbone structure with sulfonate groups. The high longitudinal stiffness of the backbone endows it with superior mechanical properties. The anions are fixed onto the polymer backbone, and cations can be freely movable under specific stimulus. Cations on sulfonic acid groups are believed to hop from one acid site to another under immigration[6].

And the internal pores in Nafion can facilitate immigration of cations in working state. The intrinsic cations in Nafion are hydrogen ions, but the cation type can be substituted with other cations through ion-exchange strategy[7]. Based on these ionic properties, Nafion has been widely studied in the fields of fuel cell, electrochemical devices, and water electrolysis[8]. The cations in the Nafion wall are hydrogen ions, and annotation has also been corrected in Supplementary Figure 4. The vacuum drying procedure is to remove residual water and methanol on the electrodes after polymerization, whereas the water molecules remained in polyelectrolyte because of barrier effect of as-formed electrode layers in the device. In order to measure the water content, we scraped off electrode materials on device and tested TGA curve of exposed polyelectrolyte. The water content is evaluated through weight loss in temperature range from 50 to 200 °C on the curve. From the TGA result in Supplementary Figure 10, it is found that water content in the as-prepared materials is 4.78%. Influence of humidity on the electrochemical performance of device has been investigated in Supplementary Figure 11, and the result indicates that specific capacitance of the device increases slightly with increasing of humidity. This is mainly because the increased humidity in environment promotes ionic transfer kinetics in polyelectrolyte[9].

The conductivity of the formed PEDOT film has been measured by multifunction digital four-probe tester, and the film shows a high electrical conductivity of 753 S cm^{-1}. In the experiment, inner and outer PEDOT films have been connected to the test circuits as two electrodes. Typically, two conductive wires were weld onto outer and inner sizes of the device, as shown in the updated Figure 5a. The influence of work temperature on electrochemical performance of the supercapacitor has been explored in Supplementary Figure 12. It was found that electrical conductivity of PEDOT electrode decreased slightly with the rise of temperature, whereas specific capacitance of the supercapacitor increased from 102 to 121 F g^{-1}. This is mainly because the higher working temperature can accelerate ionic transfer kinetics in polyelectrolyte layer while electrical conductivity of electrode does not change obviously in the temperature range.

The loading mass of the electrode and the whole of the device are measured as 16.1 mg and 70 mg, respectively. The capacitance, energy density, and power density values are based on electrode materials, and have not accounted for the electrolyte. And the gravimetric values

of electrochemical performances including the electrolyte have been provided for comparison, such as capacitance (23.5 F g^{-1} at 1 A g^{-1}), energy density (3.27 Wh kg^{-1} at 1 A g^{-1}), and power density (230.5 W kg^{-1} at 1 A g^{-1}).

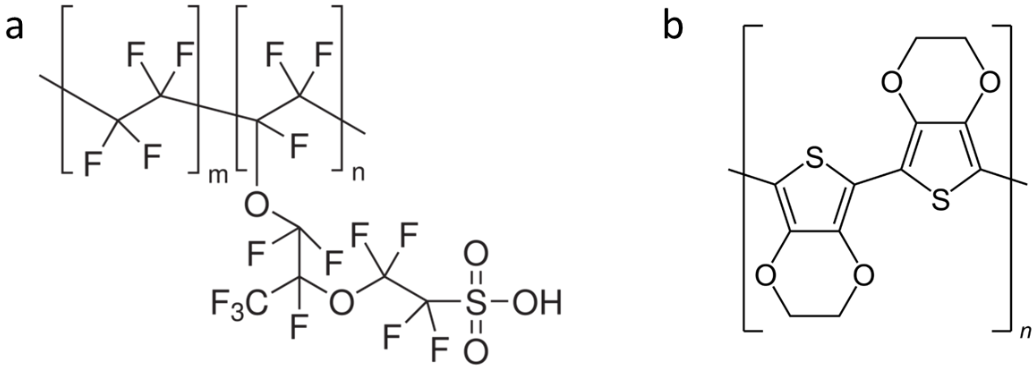

Supplementary Figure 1 Chemical structure of (a) Nafion and (b) PEDOT materials, respectively.

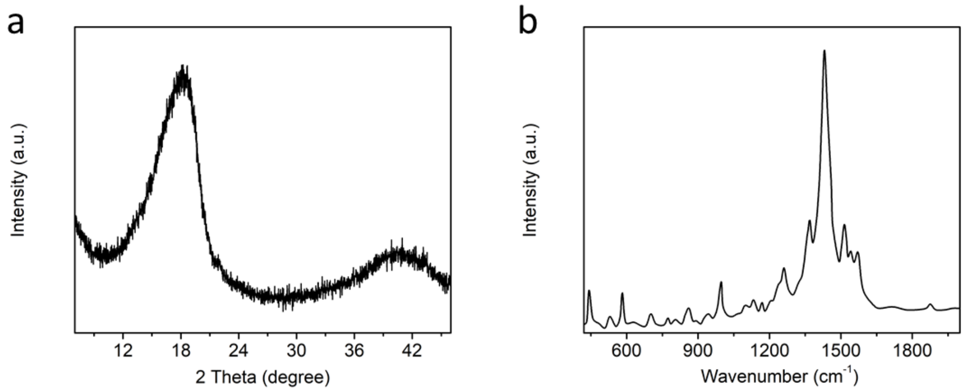

Supplementary Figure 2 (a) XRD pattern of Nafion electrolyte. (b) Raman spectra of PEDOT electrode.

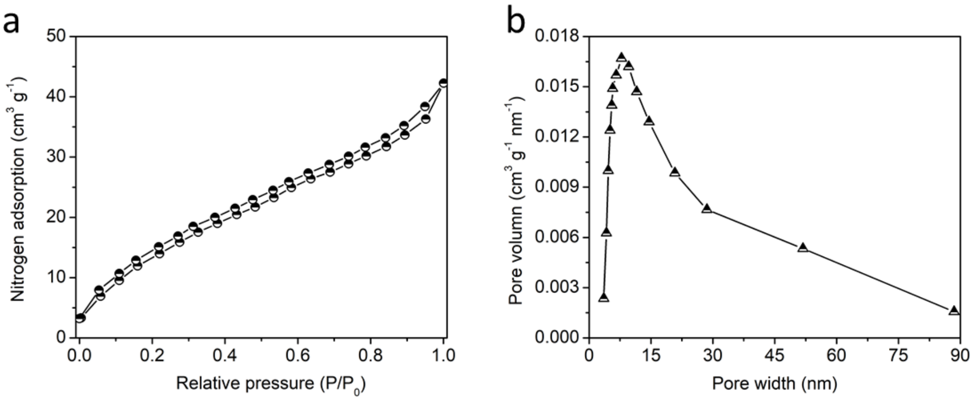

Supplementary Figure 3 (a) Nitrogen adsorption/desorption isotherm, and (b) pore-size distribution of the sample.

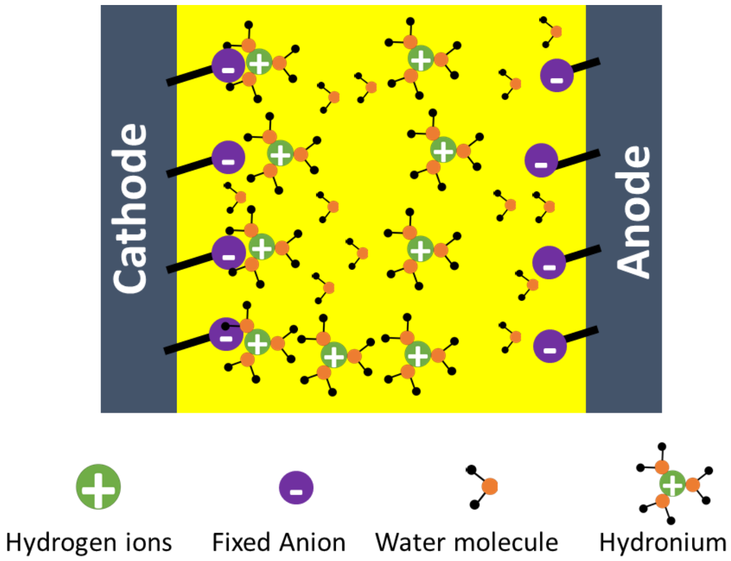

Supplementary Figure 4 Working mechanism of supercapacitors. Nafion is a kind of cation exchange resin with mobile hydrated cations and immobile anions fixed on polymer structure. Only cations immigrate to cathode during charge-discharge process and the single ion immigration mechanism improves ion mobility so as to promote the electrochemical dynamics.

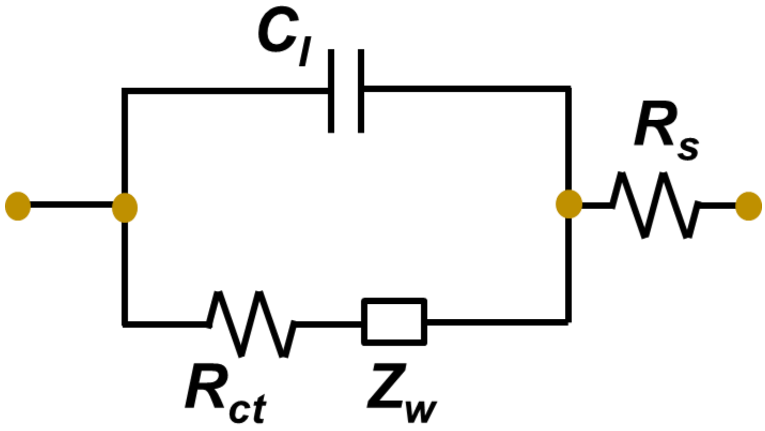

Supplementary Figure 5 The equivalent circuit model of supercapacitors.

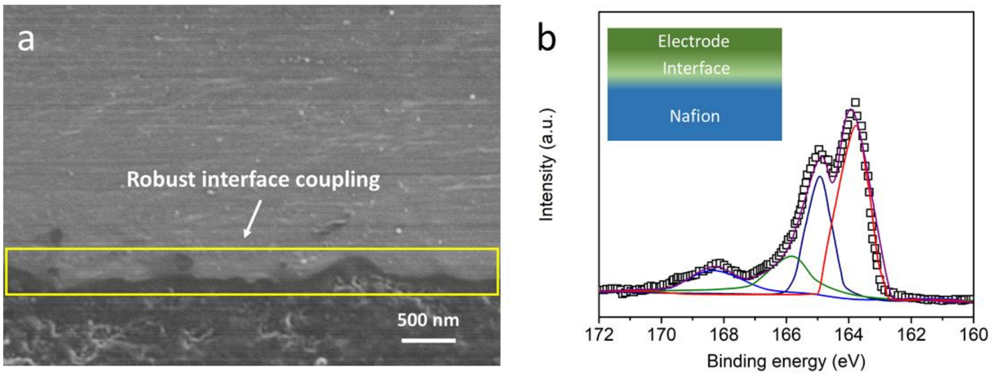

Supplementary Figure 6 a HRSEM image of device interface. **b** High-resolution S2p XPS spectra of device interface.

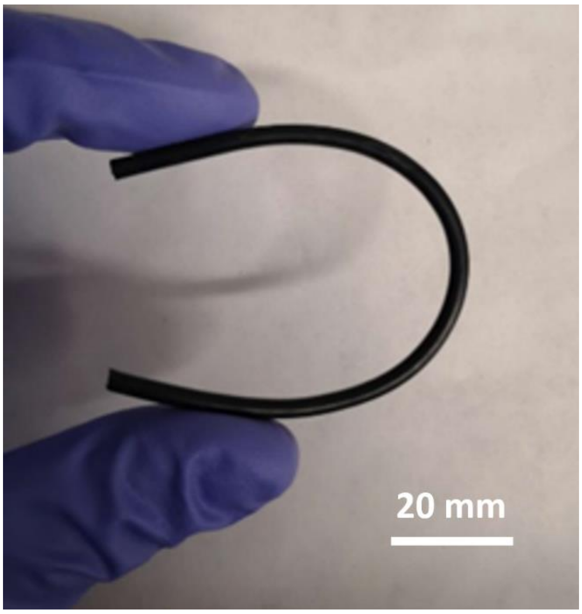

Supplementary Figure 7 Flexible device bending at the angle of 180°.

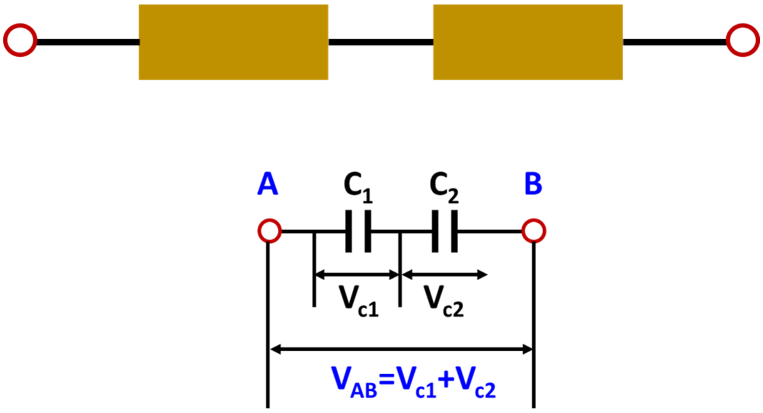

Supplementary Figure 8 Schematic and equivalent circuit diagram for two devices integrated in series.

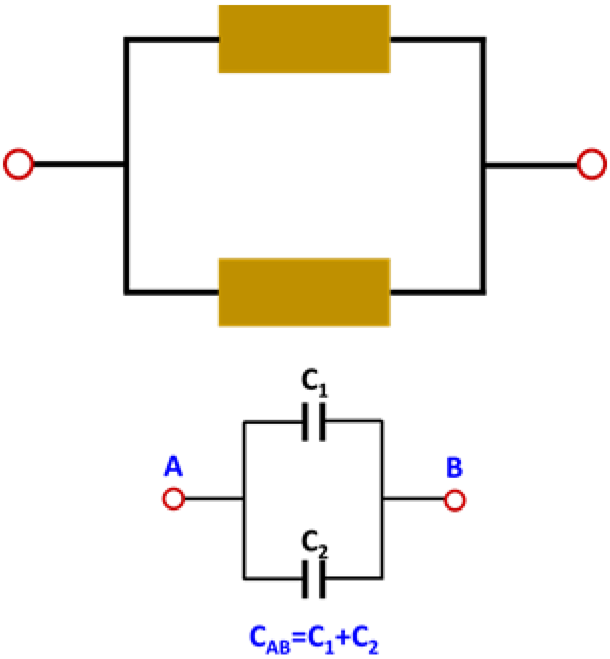

Supplementary Figure 9 Schematic and equivalent circuit diagram for two devices integrated in parallel.

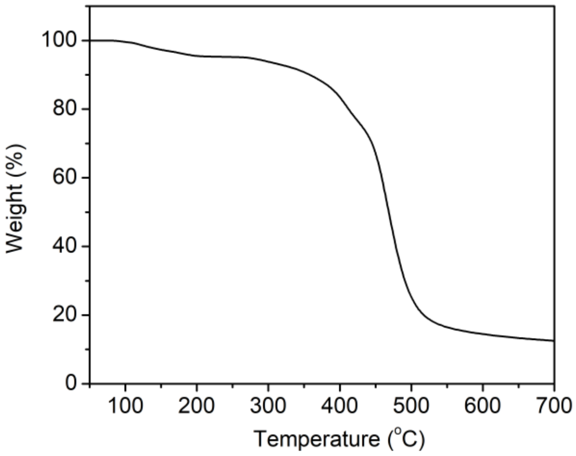

Supplementary Figure 10 TGA curve of exposed polyelectrolyte with heating rate of 10 C min^{-1} from 25 to 800 °C under nitrogen.

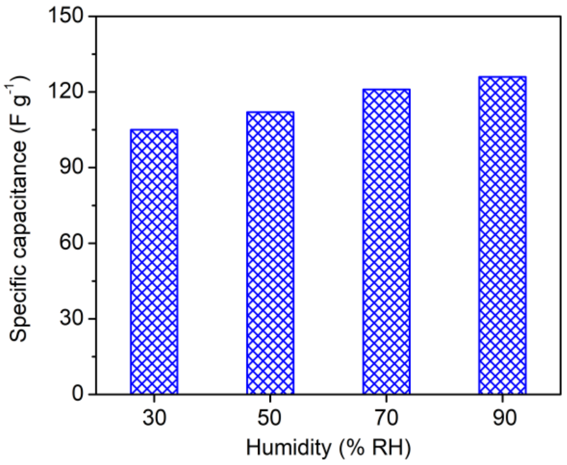

Supplementary Figure 11 Effect of humidity on specific capacitance of the device at current density of 1 A g^{-1} under potential of 1.0 V.

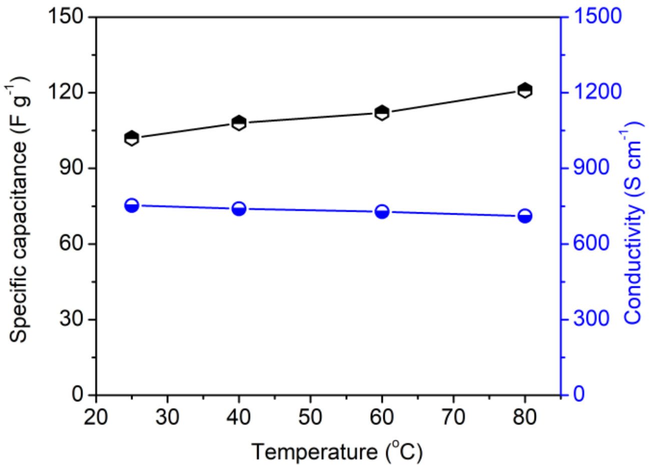

Supplementary Figure 12 The influence of working temperature on electrode conductivity and electrochemical performance of supercapacitor.

Supplementary References

1. He Y, Chen W, Li X, Zhang Z, Fu J, Zhao C*, et al.* Freestanding Three-Dimensional Graphene/MnO2 Composite Networks As Ultralight and Flexible Supercapacitor Electrodes. *ACS Nano*, **7**, 174-182 (2013).
2. Guan C, Liu X, Ren W, Li X, Cheng C, Wang J. Rational Design of Metal-Organic Framework Derived Hollow NiCo2O4 Arrays for Flexible Supercapacitor and Electrocatalysis. *Adv. Energy Mater.*, **7**, 1602391 (2017).
3. Shown I, Ganguly A, Chen L-C, Chen K-H. Conducting polymer-based flexible supercapacitor. *Energy Sci. Eng.*, **3**, 2-26 (2015).
4. Choi C, Lee JA, Choi AY, Kim YT, Lepro X, Lima MD*, et al.* Flexible supercapacitor made of carbon nanotube yarn with internal pores. *Adv. Mater.*, **26**, 2059-2065 (2014).
5. Heitner-Wirguin C. Recent advances in perfluorinated ionomer membranes: structure, properties and

applications. *J. Membr. Sci.*, **120**, 1-33 (1996).

6. Choi P, Jalani NH, Datta R. Thermodynamics and proton transport in Nafion II. Proton diffusion mechanisms and conductivity. *J. Electrochem. Soc.*, **152**, E123-E130 (2005).
7. Hickner MA, Pivovar B. The chemical and structural nature of proton exchange membrane fuel cell properties. *Fuel Cells*, **5**, 213-229 (2005).
8. Mauritz KA, Moore RB. State of understanding of Nafion. *Chem. Rev.*, **104**, 4535-4586 (2004).
9. Wee G, Larsson O, Srinivasan M, Berggren M, Crispin X, Mhaisalkar S. Effect of the Ionic Conductivity on the Performance of Polyelectrolyte-Based Supercapacitors. *Adv. Funct. Mater.*, **20**, 4344-4350 (2010).
